# Tricellulin Effect on Paracellular Water Transport

**DOI:** 10.3390/ijms20225700

**Published:** 2019-11-14

**Authors:** Carlos Ayala-Torres, Susanne M. Krug, Jörg D. Schulzke, Rita Rosenthal, Michael Fromm

**Affiliations:** Institute of Clinical Physiology/Nutritional Medicine, Medical Department, Division of Gastroenterology, Infectiology and Rheumatology, Charité—Universitätsmedizin Berlin, 12203 Berlin, Germany

**Keywords:** tricellulin, tricellular tight junction, paracellular water transport, tight epithelium, MDCK C7 cells

## Abstract

In epithelia, large amounts of water pass by transcellular and paracellular pathways, driven by the osmotic gradient built up by the movement of solutes. The transcellular pathway has been molecularly characterized by the discovery of aquaporin membrane channels. Unlike this, the existence of a paracellular pathway for water through the tight junctions (TJ) was discussed controversially for many years until two molecular components of paracellular water transport, claudin-2 and claudin-15, were identified. A main protein of the tricellular TJ (tTJ), tricellulin, was shown to be downregulated in ulcerative colitis leading to increased permeability to macromolecules. Whether or not tricellulin also regulates water transport is unknown yet. To this end, an epithelial cell line featuring properties of a tight epithelium, Madin-Darby canine kidney cells clone 7 (MDCK C7), was stably transfected with small hairpin RNA (shRNA) targeting tricellulin, a protein of the tTJ essential for the barrier against passage of solutes up to 10 kDa. Water flux was induced by osmotic gradients using mannitol or 4 and 40 kDa-dextran. Water flux in tricellulin knockdown (KD) cells was higher compared to that of vector controls, indicating a direct role of tricellulin in regulating water permeability in a tight epithelial cell line. We conclude that tricellulin increases water permeability at reduced expression.

## 1. Introduction

For a long time, the tight junction (TJ) was considered as a more or less perfect barrier to impede the paracellular permeation of solutes and water [1]. A few years after the membrane-spanning proteins occludin and the claudin family were identified to form the TJ, the first claudin forming a charge-selective channel was discovered [2] (for review see [3]), explaining how the TJ also can provide a selective pathway for the passive permeation through the paracellular pathway.

Tight junctions are organized in two ways. (i) The bicellular tight junction (bTJ) connects two neighboring cells and (ii) the tricellular tight junction (tTJ) is formed at the site where three cells meet. Ultrastructurally, at the tTJ the bicellular strands extend in the basal direction and form a central tube of assumedly 1 μm in length and 10 nm in diameter [4]. While the bTJs is comprised of the transmembrane proteins occludin and the family of claudins [5], the predominant proteins making up the tTJ are tricellulin and angulins [6].

Tricellulin is a tetraspan protein that plays a critical role in sealing the tTJ against the passage of solutes. If it is fully knocked out, the TJ structure is altered and barrier function breaks down [7]. At low expression rates as found in MDCK II cells, the tTJ opens a diffusional pathway at the tTJ for macromolecules of up to 10 kDa [8]. It would be reasonable if also small solutes could pass then, but in MDCK II cells this was not detectable. This paradox is explained by the fact that the tTJ is too rare to contribute significantly to ion permeability in low-resistance (leaky) epithelia in which the permeability of the bTJ predominates. In the high-resistance (tight) human colon cell line HT-29/B6 also an effect of the tTJ on ion permeability became significant [9]. In intestinal biopsies from patients suffering from ulcerative colitis, tricellulin expression was decreased and the passage of macromolecules was increased [10].

Angulins are unified names for three single-span proteins located at the tTJ, lipolysis-stimulated lipoprotein receptor (LSR, angulin-1), immunoglobulin-like domain-containing receptor 1 (ILDR1, angulin-2), and ILDR2 (angulin-3). Angulin-1 has been shown to recruit tricellulin to the tTJ [11] and the same effect was observed for angulin-2 and -3 [12]. Angulin-1 and -2 are expressed in a complementary way with angulin-3. In addition, angulin-1 and -2 exert a barrier-maintaining effect, while angulin-3 has a weaker effect in this regard [12].

Whether or not the TJ at all is permeable to water was highly controversial for a long time. The transcellular pathway for water via aquaporin channels was established more than 20 years ago [13] and their presence has been described in a wide range of tissues [14]. Another transcellular pathway for water is provided by the Na^+^-glucose cotransporter SGLT1 (and several other carriers) which contributes, especially in small intestinal epithelia, significantly to water transport [15].

Regarding the paracellular pathway through the bTJ, Rosenthal et al. have developed an elaborated though difficult to handle technique to measure water transport on monolayers mounted in Ussing chambers [2]. First it was demonstrated that claudin-2, so far identified as a paracellular cation channel [2], also forms a water channel [16]. Cations and water were then shown to pass through the same pore [17]. Recently, a second water channel through the bTJ was identified to be claudin-15, which also was known so far as a cation channel only [18].

With this in mind, three subtypes of channel-forming claudins can be functionally distinguished regarding their permeability to water: (i) proteins forming channels for anions but not for water (claudin-10a and -17) [19,20], (ii) proteins forming channels for cations but not for water (claudin-10b) [16], and (iii) proteins forming channels for cations and also for water (claudin-2 and -15) [16,17,18]. After it has been demonstrated that the tTJ can provide another paracellular pathway for solutes [8,9,10,21], the question arises whether or not it also contributes to water passage.

As for the tTJ, Gong et al. provided compelling evidence that water transport is increased in isolated kidney tubules in the absence of angulin-2, but is normally inhibited in presence of angulin-2 [22]. The role of tricellulin has not been elucidated so far. We hypothesize that tricellulin is a direct negative actuator of tTJ water permeability. Therefore, we aimed to characterize the effect of tricellulin expression on water transport in the tight epithelial cell line MDCK C7 and found that the water passage through the tTJ was indeed enhanced under the influence of reduced tricellulin levels.

## 2. Results

### 2.1. Characterization of Tricellulin Knockdown in MDCK C7 Cells

**Figure 1:**
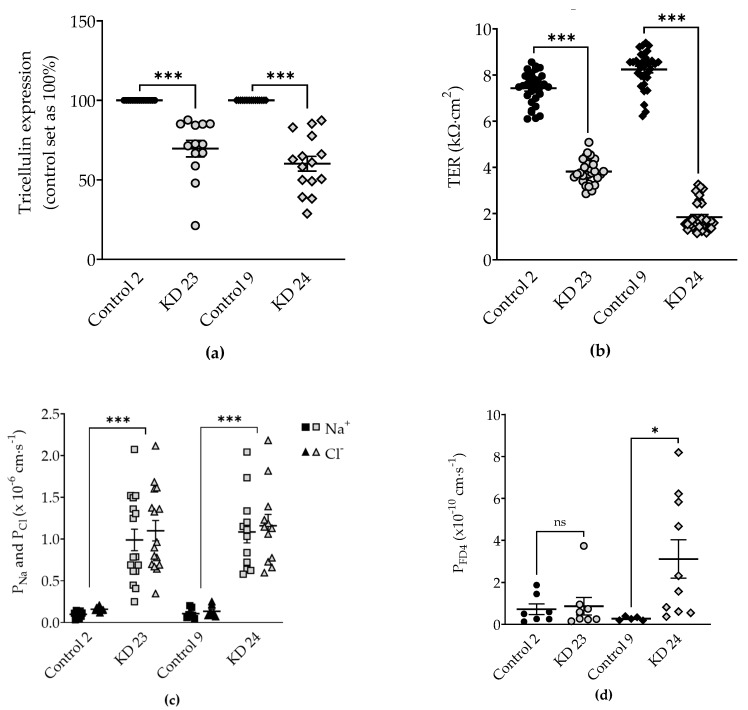
Expression and functional analysis of tricellulin knockdown in MDCK C7 cells. (**a**) Densitometric analysis of tricellulin protein expression levels in stable shTRIC transfectants (KD 23 and KD 24) in comparison to vector-transfected controls (Control 2 and Control 9). shTRIC leads to decreased tricellulin expression (*n* = 15). (**b**) Effect of tricellulin knockdown on transepithelial resistance (TER). Tricellulin knockdown decreases TER in MDCK C7 cells (*n* = 24). (**c**) Effect of tricellulin knockdown on ion permeability. Tricellulin knockdown increases Na^+^ and Cl^−^ permeability to the same extent in MDCK C7 cells (*n* = 7–16). (**d**) Effect of tricellulin knockdown on permeability for 4 kDa-FITC dextran (FD4). Permeability is increased in tricellulin KD 24 cells only (*n* = 5–10). Statistical analysis was performed using Student’s *t*-test between the KD clone and the corresponding control (KD 23 versus control 2, KD 24 versus control 9). (Symbols of (a), (b) and (d): Control 2: ●; KD 23: 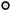; Control 9: ◆; KD 24: 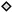. * *p* <0.05, *** *p* < 0.001, ns = not significant).

After transfection of Madin-Darby canine kidney cells clone 7 (MDCK C7) with shRNA targeting tricellulin, puromycin-resistant cell clones were screened for tricellulin knockdown by western blotting. In this study, two knockdown clones and two control clones were investigated (KD 23 and its corresponding control 2, and KD 24 and its corresponding control 9). The two KD clones showed high but different reduction in tricellulin expression: clone KD 23 showed a depression of tricellulin by 30% while clone KD 24 exhibited a drop by 40% (Figure 1a and Table 1).

The transepithelial resistance (TER) was reduced in tricellulin knockdown MDCK C7 cells (Figure 1b, Table 1). KD 24 exhibited a stronger reduction of tricellulin expression and stronger decrease in TER compared to the corresponding control than KD 23. This was due to a huge increase in Na^+^ and Cl^−^ permeability without any preference for anions or cations (Figure 1c, Table 1).

Then we examined the permeability of the four clones to fluorescein isothiocyanate-dextran 4 kDa (FITC–dextran, FD4). As a result, FD4 permeability of KD 23 was not significantly different from its control, but that of clone KD 24 exhibited a strong increase (Figure 1d, Table 1). Although clones KD 23 and KD 24 differed only gradually in reduction of tricellulin expression and TER decrease, the two chosen clones differed in macromolecule permeability.

### 2.2. Effects of Tricellulin Knockdown on Endogenous Proteins of MDCK C7 Cells

**Figure 2:**
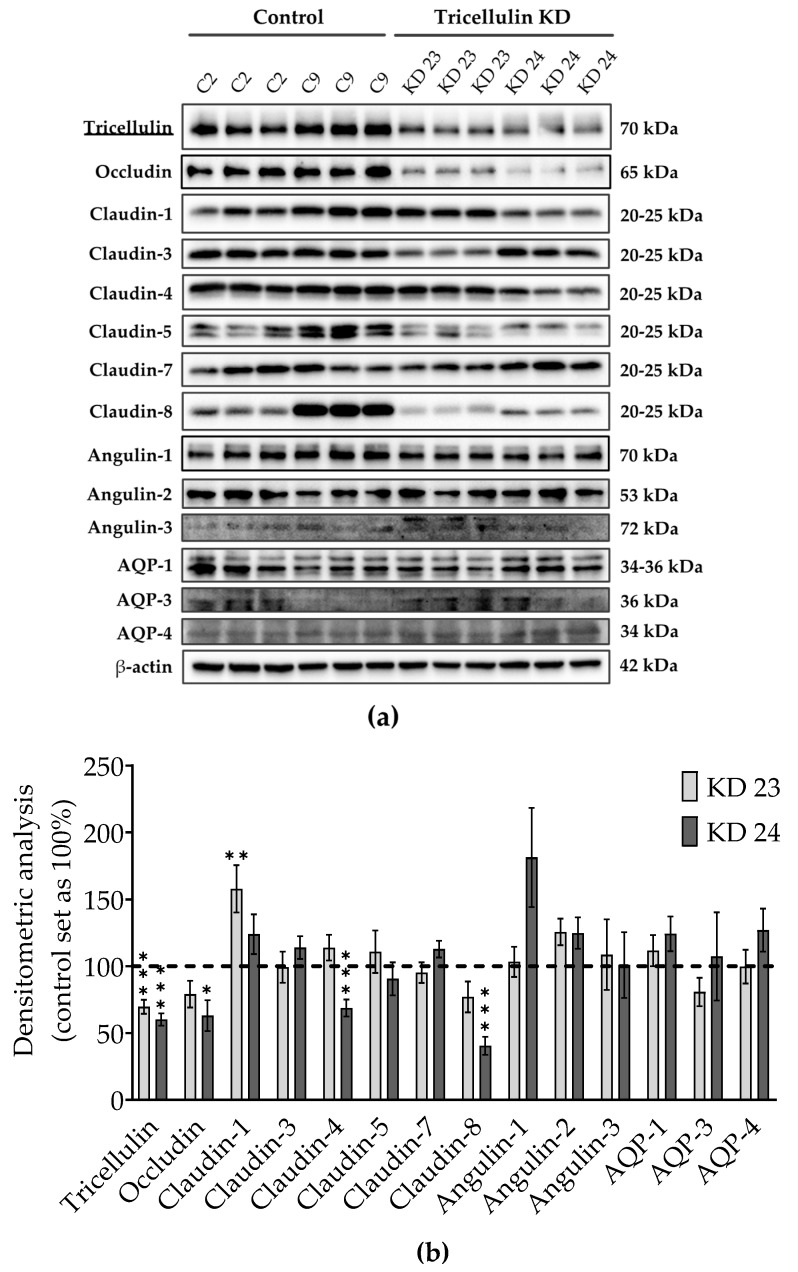
Claudin, occludin, angulin and aquaporin (AQP) expression in tricellulin knockdown MDCK C7 cells. (**a**) Representative western blots. (**b**) Densitometric analysis of protein expression levels in tricellulin knockdown clones KD 23 and KD 24 in comparison to the corresponding vector-transfected controls (*n* = 9–17, *N* = 7). β-Actin was used as an internal control for normalization to protein content. Statistical analysis was performed using Student’s *t*-test between the KD clone and the corresponding control (KD 23 versus control 2, KD 24 versus control 9). (* *p* <0.05, ** *p* < 0.01, *** *p* < 0.001).

Knockdown of one TJ protein may cause variation in other proteins, which are involved in transepithelial water transport. Therefore, we examined levels of TJ proteins, such as occludin, several claudins, the three angulins, and of aquaporin (AQP) water channels, AQP1, 3, and 4 (Figure 2a). The densitometric analysis revealed some clonal variability in TJ protein expression between the knockdown clones and the controls. In detail, claudin-1 was increased in the tricellulin KD 23, and occludin and claudin-4 and -8 were decreased in the tricellulin KD 24 (Figure 2b). Clonal variability in protein expression was also observed between both control clones and both KD clones. None of the three angulins were significantly altered. Most importantly, the tricellulin knockdown did not affect the expression of the cell membrane water channels expressed in MDCK C7 cells, AQP1, AQP3, and AQP4 (Figure 2b).

**Figure 3:**
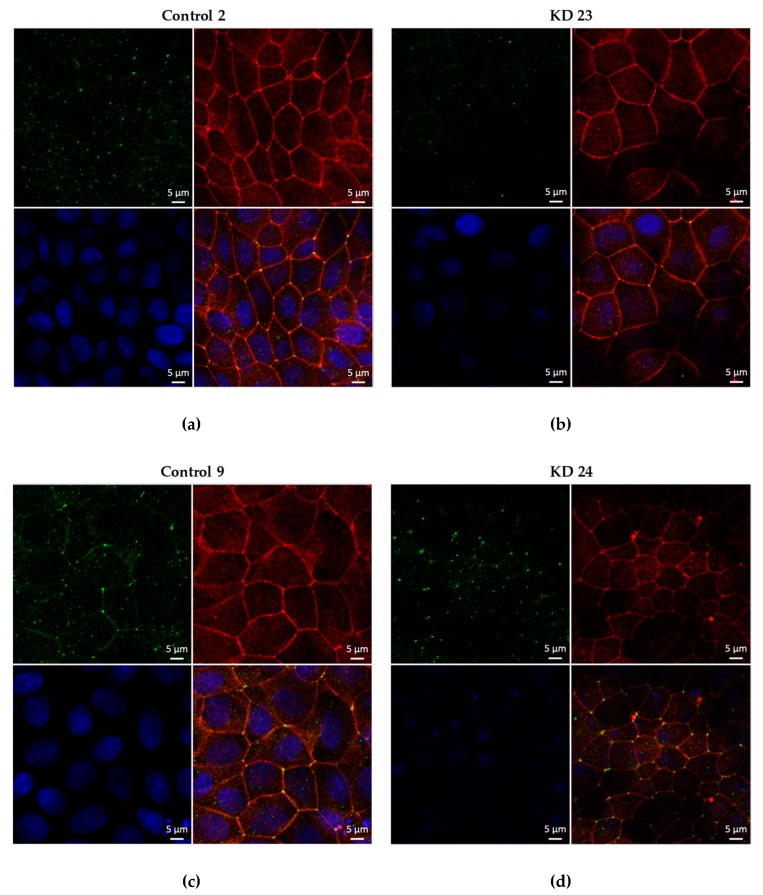
Localization of tricellulin in the four clones used throughout this study. In the KD clones, tricellulin is localized at tricellular tight junction (tTJ) sites. Tricellulin: green; occludin: red; cell nuclei (4′,6-Diamidine-2′-phenylindole dihydrochloride - DAPI): blue. MDCK C7 cells transfected with (**a**,**c**) empty lentiviral vector for shRNA expression (pLKO.1-puro) as a negative control, (**b**) pLKO.1-puro vector containing a sequence for shRNA targeting tricellulin (shRNA 23), and (**d**) pLKO.1-puro vector containing a sequence for shRNA targeting tricellulin (shRNA 24).

The proper localization of tricellulin was confirmed for the four clones by immunofluorescence confocal laser-scanning microscopy. Occludin served as a TJ marker (Figure 3).

### 2.3. Effect of Tricellulin Knockdown on Transepithelial Water Transport in MDCK C7 Cells

**Figure 4:**
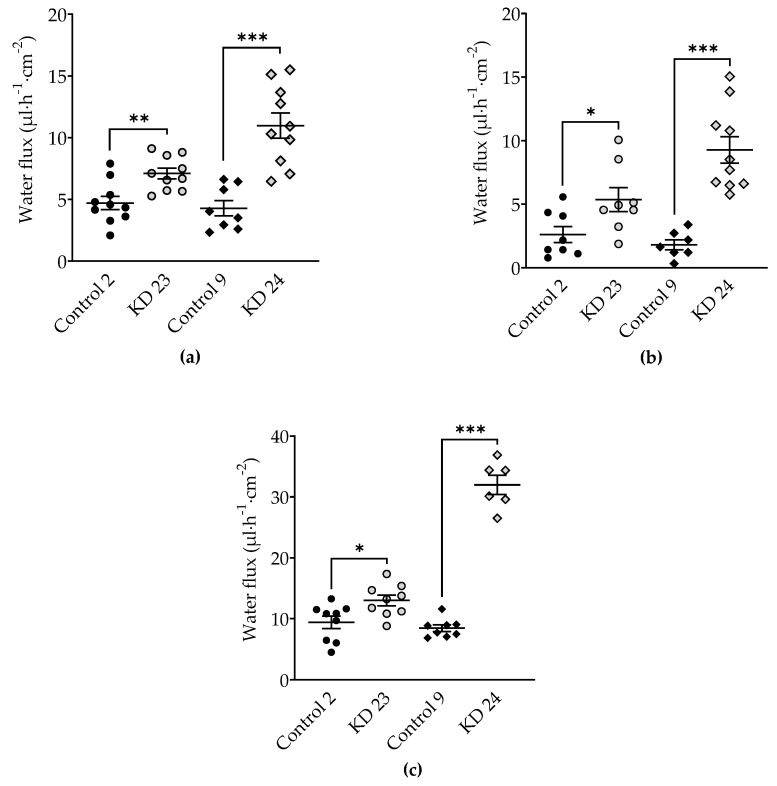
Water flux in tricellulin knockdown MDCK C7 cells stimulated by an osmotic gradient on the apical side of the cell layer. Water flux induced by a gradient of (**a**) 100 mM mannitol (*n* = 8–10), (**b**) 37 mM 4 kDa-dextran (*n* = 8–10) and (**c**) 100 mM 4 kDa-dextran (*n* = 6–9) was increased in both tricellulin knockdown clones, with the stronger increase in clone KD 24 with the higher reduction of tricellulin expression. Statistical analysis was performed using Student’s *t*-test between the KD clone and the corresponding control (KD 23 versus control 2, KD 24 versus control 9). (Control 2: ●; KD 23: 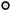; Control 9: ◆; KD 24: 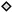. * *p* <0.05, ** *p* < 0.01, *** *p* < 0.001).

To analyze the effect of tricellulin knockdown on water permeability of MDCK C7 cells, water flux was measured after induction with osmotic gradients produced by 100 mM mannitol, by 37 mM 4 kDa-dextran, or by 100 mM 4 kDa-dextran (Figure 4). These concentrations produced measured osmolality gradients of 100 mOsm for 100 mM mannitol and for 37 mM 4 kDa-dextran, and of 900 mOsm for 100 mM 4 kDa-dextran.

Tricellulin KD 23 as well as KD 24 exhibited increased water fluxes compared with their respective vector control clones, however the change obtained by KD 24 was larger under all conditions (Figure 4, Table 1). In KD 24, the increase under a mannitol-induced osmotic gradient was 2.6-fold (Figure 4a), under a gradient induced by 37 mM 4 kDa-dextran it was 5.2-fold (Figure 4b) and under a gradient induced by 100 mM 4 kDa-dextran the increase was 3.8-fold (Figure 4c), compared to 1.5-fold, 2.1-fold, and 1.4-fold in KD 23, respectively.

**Figure 5:**
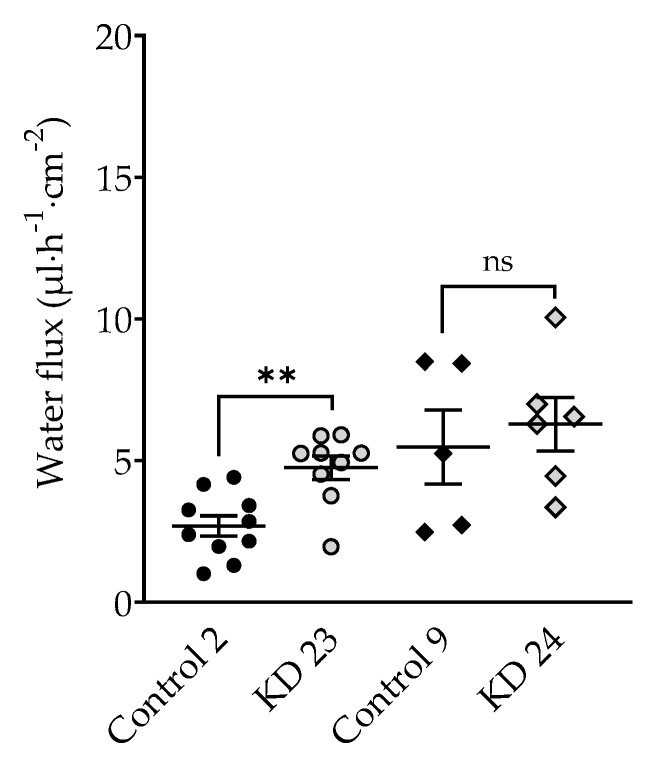
Water flux in tricellulin knockdown MDCK C7 cells stimulated by an osmotic gradient of 5.5 mM 40 kDa-dextran (100 mOsm) on the apical side of the cell layer. No effect could be observed in tricellulin KD 24 cells, whereas in KD 23 with the higher tricellulin expression, the increase was in the same order of magnitude as in the presence of 100 mM (100 mOsm) mannitol. Statistical analysis was performed using Student’ *t*-test between the KD clone and the corresponding control (KD 23 versus control 2, KD 24 versus control 9). (Control 2: ●; KD 23: 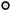; Control 9: ◆; KD 24: 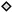. ** *p* ≤ 0.01, ns = not significant).

In a parallel same series of experiments, a 5.5 mM 40 kDa-dextran gradient was applied which produced a measured osmolality gradient of 100 mOsm. In the presence of 100 mOsm 40 kDa-dextran, water flux was lower than in the presence of 100 mOsm mannitol. The 40 kDa-dextran gradient did not significantly change water flux in tricellulin KD 24 compared to control, whereas water flux in KD 23 was increased (Figure 5, Table 1). This finding indicates that the water flux through the tTJ was inhibited in the presence of 40 kDa-dextran in the clone with the strong reduction of tricellulin.

## 3. Discussion

This study shows that tricellulin downregulation in the tight epithelial cell line MDCK C7 resulted in an increased ion permeability, as shown by the reduction of TER and, dependent on the reduction of tricellulin, in an increased macromolecule permeability. Additionally, a decrease in tricellulin expression is associated with an increased transepithelial water flux. Thus, tricellulin is able to regulate osmotically-induced transepithelial water flux in a tight epithelium. This regulation occurs in a reciprocal way, i.e., lowered tricellulin expression causes higher water fluxes.

### 3.1. Effect of Tricellulin Knockdown on Macromolecule Passage

The MDCK cell line is derived from canine kidney cells and common strains feature properties of either proximal (MDCK II, MDCK C11) or distal nephron (MDCK I, MDCK C7) [23,24].

Transfection of the tight epithelial cell line MDCK C7 with shRNA targeting tricellulin resulted in a reduction of this protein in the tTJ. In agreement with former experiments on cell cultures [10], the reduction in the expression of tricellulin resulted in a lowered transepithelial resistance and the development of a pathway for macromolecules. Complete knockout, however, leads to a disorganization of not only tTJs but also bTJs and complete breakdown of barrier parameters [7]. This is of clinical importance in genetic defects in tricellulin causing hearing loss [25], however, for studies focused selectively on the tTJ, graded knockdown approaches that reduce tricellulin only in part are suitable.

In addition, the permeability to the paracellular marker 4 kDa-dextran was increased by tricellulin knockdown as normally this protein is responsible for blocking the passage of macromolecules with diameters from 1.3 to 4.6 nm (0.9 to 10 kDa) [8]. This is consistent with the present data showing that in tight epithelial cell lines a downregulation of tricellulin strongly regulates the permeability for 4 kDa-macromolecules. It might be surprising that a moderate knockdown of tricellulin by 40% already leads to five times higher permeability for macromolecules. This is explained by the fact that normally the permeability for macromolecules is extremely low (in the order of 10^−12^ cm/s) so that even a slight opening of a pathway for macromolecules causes a significant effect [21]. Furthermore, the reduction by 30% (KD 23) had no significant effect on macromolecule permeability, however, did have an effect on ion permeability, indicating that there is already a significant impairment of the tTJ barrier which however is only affecting small solutes, while 4 kDa-dextran is still not able to pass. This leads to the assumption that the varying range of tricellulin expression may lead to different effects. The limit of macromolecule passage seems to range between a 30% to 40% reduction of tricellulin, while for small solutes the regulation is stricter.

### 3.2. Effects of Tricellulin Knockdown on Other Proteins of MDCK C7 Cells

Immunoblot analysis with an anti-tricellulin antibody demonstrated a clear reduction of tricellulin expression (Figure 2a). Nevertheless, the generated tricellulin knockdown clones compared with the controls showed clonal variation in other TJ proteins which reached significance for occludin, claudin-1, claudin-4, and claudin-8 (Figure 2a,b). The clonal variation also concerns the two control clones, here the differences are most prominent for claudin-1, claudin-5, and claudin-8. These claudins seem to have no effect on paracellular water transport, since the water transport of the two control clones did not show any difference. In the tricellulin knockdown clones, the expression of occludin, claudin-1, claudin-4, and claudin-8 was changed compared to the corresponding vector control. Considering the functional aspects of the variations it can be said that occludin, which is downregulated only in one of the tricellulin knockdown clones, is assumed to have no effect on TJ barrier function, either genuinely or through compensation of its downregulation by other tight junction proteins [26,27,28]. All claudins present in MDCK C7 cells are known to behave as barrier formers, acting in concert and compensating their respective up- and down-regulation [29]. Ion and water channel-forming claudins can be disregarded as they are not genuinely present in MDCK C7 cells. A reduced expression of claudin-4 and claudin-8 and an increased expression of claudin-1 was found in one of the knockdown clones, respectively. However, claudin-4 is described to be dispensable for the barrier properties of TJs in wild-type MDCK II cells [30]. Furthermore, claudin-1 and claudin-4 were shown to be dispensable for water barrier formation in human submerged keratinocyte cultures [31]. Therefore, we feel safe to conclude that the observed clonal variation of the claudins in sum is balanced and provides a constant barrier function of the bTJ. Most importantly, the three angulins as well as the aquaporins AQP-1, -3, and -4 did not significantly change. Thus, the changes in ion, macromolecule and water permeability found in the knockdown clones can be assumed to be exclusively due to the reduced tricellulin expression in the tTJ.

That the knockout of a tTJ-related protein, angulin-2, increases water transport was shown in elegant experiments by Gong et al. on isolated mouse kidney tubules [24]. The authors suggest that the barrier function of angulin-2 derives from a trans-interaction between its extracellular immunoglobulin-like domain. All three angulins are known to be able to recruit tricellulin to the tTJ [11]. It is beyond the scope of the present study but will be subject of future investigation, to experimentally clarify whether angulins mediate a direct effect on water permeability independent of tricellulin, or angulins act indirectly via tricellulin regulation.

### 3.3. Water Transport as Driven by Different Osmotic Gradients

In other studies from this lab using the method of generating water flux by an osmotic gradient e.g., by mannitol, this solute was impermeable and could not pass the TJ by itself [16,17,18,19,20]. In the present study, for the first time this was different and more complicated. In the tricellulin KD clones the tTJ central tube is opened, and through this pathway mannitol can diffuse following its concentration gradient. This has two consequences: First, in the course of the experiment, mannitol may be diluted and its gradient is reduced. However, this will be below significance, because the amount of mannitol in the 9 mL bath solution is high compared to the amount diffusing through the tTJ. Second and more disturbing, mannitol would diffuse through the central tube in the opposite direction to the presumed water flux. As long as a molecule like mannitol is small compared to the diameter of the central tube, this may not have a large effect. As this mechanism may alter water transport rates at least slightly, we decided not to calculate water permeability coefficients in this study and present data as fluxes only.

In order to investigate the side effect of mannitol diffusion, we compared data obtained with 100 mM mannitol with those obtained with larger molecules, 37 mM 4 kDa- and 5.5 mM 40 kDa-dextran. For this, dextran concentrations were chosen which all produced an osmolality of 100 mOsm, as used for mannitol. This resulted in lower nominal concentrations due to a known water-sequestering effect of some macromolecules [32].

It turned out that gradients of 100 mM mannitol and 37 mM 4 kDa-dextran produced comparable water fluxes and that an elevated 4 kDa-dextran concentration (100 mM resulting in roughly 900 mOsm) led to higher water fluxes (see Table 1). Since these numbers satisfactorily fit together this indicates the reliability of the methods used.

The most valuable results should have been obtained with an osmotic gradient obtained by 5.5 mM 40-dextran (measured osmolality 100 mOsm). Surprisingly, this was not the case: In the presence of 40 kDa-dextran, water flux was reduced in the control clones as well as in the knockdown clones compared to water fluxes in the presence of mannitol with the same osmolality. An induction of water flux by a gradient with 40 kDa-dextran did not produce significantly changed water fluxes in the KD 24 clone with the stronger tricellulin reduction, whereas KD 23 showed an increased water flux compared to the control clone. As an explanation for this effect, the sizes of the molecule should be related to that of the tTJ central tube. The hydrodynamic radius of 40 kDa-dextran reportedly amounts 6.6 nm [33] while the diameter of the tTJ central tube was assumed to be 10 nm [4]. We suggest that due to its molecular size the 40 kDa-dextran only barely fits through the central tube or even clogs its entrance so that water is hindered to pass. Thus, water flux was reduced in all clones compared to mannitol-induced fluxes. The strongest reduction was observed in the KD 24 clone with the lowest tricellulin expression and possibly the largest diameter of the tTJ central tube. These assumptions are supported by the findings of Krug et al. that the tTJ central tube at low tricellulin levels allows for passage of molecules of 10 kDa but not of 20 kDa [8]. This reasoning finds its equivalent in experiments dealing with the bTJ, where a NaCl gradient was used to drive water flux through the claudin-15 channel. Under this condition, claudin-15-mediated water flux was inhibited by Na^+^ diffusion in the opposite direction [18].

### 3.4. Effect of Tricellulin Knockdown on Transepithelial Water Transport

For the full line of experiments, two tricellulin KD clones were selected, which differ in the grade of tricellulin reduction, namely by 30% (clone KD 23) and by 40% (clone KD 24) compared to the respective controls. While both KD clones caused a decrease in TER, only KD 24 produced an increase in permeability to a 4 kDa-macromolecule and, most importantly, an increase of water flux driven by different osmotic gradients as discussed above.

**Figure 6:**
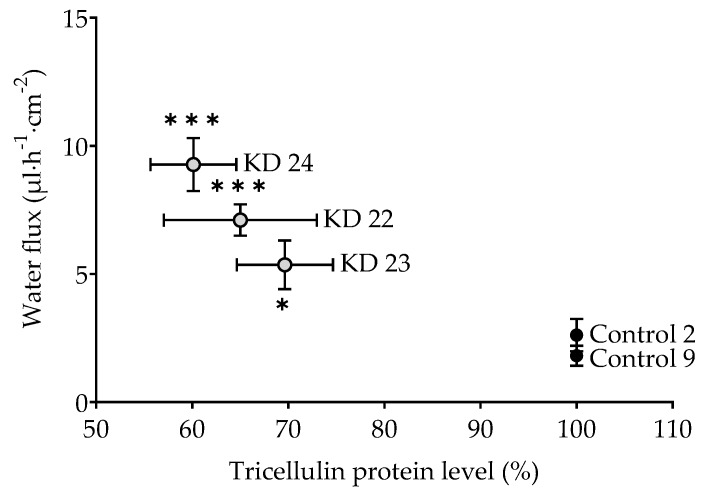
Water flux as a function of the level of tricellulin expression in MDCK C7 cells driven by an osmotic gradient of 37 mM 4 kDa-dextran (100 mOsm). Tricellulin KD 22 (*n* = 7) is an additional KD clone that was tested only for this purpose (shRNA 22: TRCN0000072635, NM_144724.1-989s1c1, Sigma-Aldrich, Schnelldorf, Germany) and compared with Control 9. Statistical analysis was performed using Student’s *t*-test between the KD clone and the corresponding control (KD 23 versus control 2, KD 24 versus control 9). In case of the KD 22 and KD 24 and their control 9, the Bonferroni-Holm adjustment for multiple testing was applied. (* *p* < 0.05, *** *p* < 0.001).

The question arises whether this is an all-or-nothing effect. If yes, one would postulate a threshold of tricellulin depression somewhere between 30% and 40%. In order to have closer look, an additional experiment was performed employing a clone with tricellulin reduced by 35% (clone KD 22). Being aware that this is based on three points only, resulting water flux did appear to correlate with the tricellulin expression, suggesting that the effect of tricellulin KD depression is gradual, starting at the level found for the controls (Figure 6).

## 4. Materials and Methods

### 4.1. Cell Culture, Transfection, and TER Measurement

The kidney cell line MDCK C7 (RRID: CVCL_0423) exhibits a high transepithelial resistance and other basic properties making it an excellent model of a tight epithelium. For stable tricellulin knockdown, MDCK C7 cells were transfected with pLKO.1-puro vector containing a sequence for shRNA targeting tricellulin (shRNA 23: TRCN0000072633, NM_144724.1-2011s1c1; and shRNA 24: TRCN0000072634, NM_144724.1-1097s1c1; Sigma-Aldrich, Schnelldorf, Germany) or pLKO.1-puro empty vector as a negative control (Sigma-Aldrich, Schnelldorf, Germany).

The transfected cells were incubated at 37 °C and 5% CO_2_ in sterilization incubators held. For the cultivation of the cells sterile culture vessels made of plastic were used. The cells were cultured in a nutrient medium Earl’s salts MEM (minimal essential medium) supplemented with 10% FCS as well as 100 U/mL penicillin/100 μg/mL streptomycin and 1.5 µg/mL of puromycin. Every second to third day the medium was changed. Consumption of nutrients was also visible due to a color change of the medium which contained a detecting pH indicator.

For protein quantification, water flux measurements and electrophysiological studies, cell monolayers were cultured on porous culture plate inserts (Millicell PCF filters, pore size 0.4 µm, effective area 0.6 cm^2^, Millipore GmbH, Schwalbach, Germany) for 7–9 days before they were used for experiments.

Transepithelial resistance (TER) was measured at 37 °C using chopstick electrodes (STX2, World Precision Instruments, Friedberg, Germany). Electrodes were reproducibly positioned by a semi-automatic motor-driven device and signals were processed by a low-frequency clamp (both own design). The resistances of the bathing solution and the blank filter support were subtracted from measured values, which finally were converted to Ω cm^2^.

### 4.2. Western Blot Analysis

Cells grown on culture-plate inserts were scraped and homogenized in total lysis buffer containing 10 mM of Tris, 150 mM of NaCl, 0.5% of Triton X-100, and 0.1% of sodium dodecyl sulphate (SDS), and protease inhibitors (complete^TM^ ethylenediaminetetraacetic acid (EDTA)-free; Roche, Basel, Switzerland). The samples were incubated 2 h at 4 °C (vortexed every 20 min), and then centrifuged at 11,000× *g* during 20 min. The pellet was discarded and the protein concentration in the supernatant was determined by the BCA (bicinchoninic acid) method (reagents were purchased from Pierce (Perbio Science, Bonn, Germany)) and quantified with a plate reader (Tecan Deutschland, Crailsheim, Germany). Aliquots between 10 and 15 µg protein samples were separated by 12% SDS-polyacrylamide gel electrophoresis and then transferred to a PVDF membrane (Perkin Elmer, Rodgau, Germany) for detection of tight-junction-associated marvel proteins (TAMP), claudins, angulins and AQPs. After blocking for 2 h in 1% PVP-40 and 0.05% Tween-20, membranes were incubated overnight with primary antibodies specific for tricellulin (Abfinity, Invitrogen), occludin (Invitrogen), angulin-1, -2, and -3 (Sigma-Aldrich, Germany), claudin-1, -3, -4, -5, -7, and -8 (Invitrogen), and AQP-1, -3, and -4 (Santa Cruz, Heidelberg, Germany). After removing the first antibody and three washing steps, the membranes were incubated 2 h with the second peroxidase-conjugated antibody (anti-mouse or anti-rabbit) in 2% of milk powder prepared in TBST 1X. For detection of the chemiluminescence signal induced by addition of Lumi-LightPLUS western blotting kit (Roche) a Fusion FX7 (Vilber Lourmat, Eberhardzell, Germany) were used. Densitometric analysis was performed with quantification software (Image Studio™ Lite, LI-COR Biosciences, Lincoln, Nebraska USA). Equal protein loading in each lane was verified by comparison with signals for β-actin (Sigma-Aldrich). For western blot analysis, lysates of at least three individual cell cultures were used and one representative experiment is shown.

### 4.3. Immunofluorescent Staining

Immunofluorescence studies were performed on culture-plate inserts. Confluent monolayers were rinsed with phosphate-buffered saline (PBS), fixed with 4% paraformaldehyde for 20 min and permeabilized for 10 min with PBS containing 0.5% (*v/v*) Triton X-100. To block non-specific binding sites, cells were then incubated in PBS containing 1% (*w/v*) BSA and 5% (*v/v*) goat serum (blocking solution; Biochrom) for 60 min. All subsequent washing procedures were performed with this blocking solution. After blocking, cells were incubated 60 min at room temperature with primary antibodies for tricellulin (Abfinity, Invitrogen; 1:250) and occludin (Invitrogen, 1:250), followed by washing steps and incubation during 60 min at room temperature with the respective secondary antibodies (Alexa Fluor 488 goat anti-rabbit and Alexa Fluor 594 goat anti-mouse, each 1:500; Molecular Probes MoBiTec) and 4′,6-diamidino-2-phenylindole (1:1000). Images were obtained with a confocal laser-scanning microscope (LSM 780, Zeiss, Jena, Germany) and processed using ZEN software (Zeiss, Oberkochen, Germany).

### 4.4. Measurement of 4 kDa-FITC-Dextran Flux

Flux studies were performed in conventional Ussing chambers under voltage clamp conditions. Dextran flux was measured in 5 mL circulating Ringer’s containing 37 mM unlabeled 4 kDa-dextran on the apical side. After addition of 100 μL of 37 mM FITC-labeled dialyzed dextran (4 kDa-FITC-dextran; Sigma-Aldrich) to the apical bath, basolateral samples (200 μL) were collected at 0, 20, 40, 60, 80, 100, and 120 min. Tracer fluxes were determined from FITC-dextran samples, which were measured with a fluorometer at 520 nm (Spectramax Gemini, Molecular Devices, Ismaning, Germany). Dextran permeability was calculated from P = J/Δc with P = permeability (cm s^−1^), J = flux (mol h^−1^ cm^−2^) and c = concentration (mol/L).

### 4.5. Dilution Potential Measurements

Dilution potential measurements for the determination of ion permeabilities were performed in Ussing chambers modified for cell-culture inserts. Water-jacketed gas lifts kept at 37 °C were filled with 10 mL circulating fluid on each side. The bathing solution contained (in mM) 119 NaCl, 21 NaHCO_3_, 5.4 KCl, 1.2 CaCl_2_, 1 MgSO_4_, 3 HEPES, and 10 D(+)-glucose, and was gassed with 95% O_2_ and 5% CO_2_ to ensure a pH value of 7.4. All experimental data were corrected for the resistance of the empty filter and the bathing solution. Dilution potentials were measured with modified bathing solution on the apical or basolateral side of the epithelial monolayer. In the modified bathing solution, NaCl was iso-osmotically replaced by mannitol. The ratio of P_Na_ and P_Cl_ and the absolute permeabilities for Na^+^ and Cl^−^ were calculated as described before [19].

### 4.6. Measurement of Transepithelial Water Transport

Water flux measurements were performed using a modified Ussing chamber with two glass tubes instead of the gas lifts as described before [16,17,18]. Throughout these experiments, transepithelial resistance (TER, Ω·cm^2^), short-circuit current (I_SC_, μA·cm^−2^), and transepithelial voltage (mV) were recorded routinely. Resistances of bathing solution and blank filter support were measured prior to each experiment and subtracted. The stability of TER served as an indicator of cell viability.

Cell filters were mounted in Ussing chambers and perfused with HEPES-buffered solution with the following composition (in mM): 144.8 NaCl, 2.4 Na_2_HPO_4_, 0.6 NaH_2_PO_4_, 5.4 KCl, 1.2 MgCl_2_, 1.2 CaCl_2_, 10.6 HEPES, and 10 D(+)-glucose. The pH value of the perfusion solution was pH 7.4. A rotary pump ensured constant circulation of the perfusion solution (4.0 mL·min^−1^) and thus a fast fluid exchange in both hemichambers (volume 500 μL) to avoid effects of unstirred layers on water permeability. Water flux was induced by a transepithelial osmotic gradient: (i) 100 mM mannitol, (ii), 37 mM 4 kDa-dextran, (iii) 100 mM 4 kDa-dextran, or (iv) 5.5 mM 40 kDa-dextran. The solution was added in the apical compartment of the Ussing chamber.

The osmolality of the perfusion solutions (mosmol/kg, abbreviated mOsm) was determined using a Vapor Pressure Osmometer (5100B, Wescor, Logan, UT). The osmolality of the HEPES-buffered solution for water flux measurements was 287.4 ± 5.7 mOsm (*n* = 15). The solutions for measuring water flux ((i) 100 mM mannitol, (ii), 37 mM 4 kDa-dextran, (iii) 100 mM 4 kDa-dextran or (iv) 5.5 mM 40 kDa-dextran) increased the osmolality to 388.2 ± 3.2 mOsm (*n* = 5), 399.4 ± 2.4 mOsm (*n* = 10), 877.8 ± 13.7 mOsm (*n* = 5), and 390.2 ± 2.5 mOsm (*n* = 5), respectively. Molecular diameters of the dextrans were estimated using the equation: d_(Å)_ = 0.215·(MW^0.587^).

The fluid level in both glass tubes was monitored by a visual system ColorView XS (Olympus Soft Imaging Solutions GmbH, Munster, Germany), at time 0 min and with intervals of 10 min over a period of 120 min. Transepithelial water flux, given as flux per square centimeter and hour, was calculated after special calibration from the difference between the menisci at the registration times. Fluxes directed from the basolateral to the apical compartment were defined as positive flux.

### 4.7. Statistical Analysis

Data are expressed as mean values ± SEM (standard error of the mean), indicating *n* as the number of single measurements, and *N* as the number of independent experiments, which means independent seeding of cells. Statistical analysis was performed using Student’s *t*-test between the KD clone and the corresponding control (KD 23 versus control 2, KD 24 versus control 9). In case of the KD 22 and KD 24 data of Figure 6, the Bonferroni–Holm adjustment for multiple testing was applied. *p* < 0.05 was considered significant (* *p* < 0.05, ** *p* < 0.01, *** *p* < 0.001).

## 5. Conclusions

This study analyzed the contribution of the tTJ to water permeability as regulated by its prominent protein tricellulin. Our results demonstrate that tricellulin KD in MDCK C7 cells, which lack the bTJ cation and water channels claudin-2 and -15, results in an increase in transepithelial water transport. This water flux appears to be negatively correlated with the tricellulin expression level. We conclude that in a tight epithelial cell line, tricellulin contributes to regulate water flux through the paracellular pathway.

## Figures and Tables

**Table 1:** Characteristics of MDCK C7 tricellulin knockdown clones and the corresponding controls. Two tricellulin knockdown clones and their corresponding controls were analyzed in this study (Control 2 and KD 23, Control 9 and KD 24). Data of tricellulin expression have been obtained by densitometric analysis of western blots using β-actin for normalization. Data of Na^+^/Cl^−^ permeability and absolute permeabilities for Na^+^ and Cl^–^ (P_Na_, P_Cl_) were obtained from dilution potential measurements in the Ussing chamber. Water flux measurements were performed in a modified Ussing chamber with water flux induced by different osmotic gradients. Significances refer to respective controls. *n* number of experiments, * *p* ≤ 0.05, ** *p* ≤ 0.01, *** *p* ≤ 0.001.

		Control 2	KD 23	Control 9	KD 24
**Tricellulin Expression (%)**		100 (*n* = 15)	69.9 ± 4.8 *** (*n* = 13)	100 (*n* = 15)	60.1 ± 4.6 *** (*n* = 15)
TER (kΩ·cm^2^)		7.4 ± 0.2 (*n* = 24)	3.8 ± 0.2 *** (*n* = 24)	8.2 ± 0.2 (*n* = 24)	1.8 ± 0.1 *** (*n* = 24)
P_FD4_ (×10^−10^ cm·s^−1^)		0.72 ± 0.23 (*n* = 7)	0.87 ± 0.39 (*n* = 8)	0.28 ± 0.03 (*n* = 5)	3.11 ± 0.86 * (*n* = 10)
P_Na_ (×10^−6^ cm·s^−1^)		0.10 ± 0.01 (*n* = 9)	0.99 ± 0.13 *** (*n* = 16)	0.11 ± 0.02 (*n* = 7)	1.08 ± 0.13 *** (*n* = 12)
P_Cl_ (×10^−6^ cm·s^−1^)		0.16 ± 0.01 (*n* = 9)	1.10 ± 0.12 *** (*n* = 16)	0.14 ± 0.03 (*n* = 7)	1.16 ± 0.14 *** (*n* = 12)
P_Na_/P_Cl_		0.81 ± 0.05 (*n* = 9)	0.94 ± 0.04 (*n* = 16)	0.85 ± 0.07 (*n* = 7)	1.02 ± 0.03 (*n* = 12)
Water flux (µl·h^−1^·cm^−2^)	100 mM mannitol (100 mOsm)	4.7 ± 0.5 (*n* = 10)	7.1 ± 0.4 ** (*n* = 10)	4.3 ± 0.6 (*n* = 8)	11.0 ± 1.0 *** (*n* = 10)
	37 mM 4 kDa-dextran (100 mOsm)	2.6 ± 0.7 (*n* = 8)	5.4 ± 0.8 * (*n* = 8)	1.8 ± 0.5 (*n* = 7)	9.3 ± 1.0 *** (*n* = 10)
	100 mM 4 kDa-dextran (900 mOsm)	9.4 ± 1.0 (*n* = 9)	13.0 ± 0.8 * (*n* = 9)	8.4 ± 0.5 (*n* = 8)	32.0 ± 1.4 *** (*n* = 6)
	5.5 mM 40 kDa-dextran (100 mOsm)	2.7 ± 0.3 (*n* = 10)	4.7 ± 0.6 ** (*n* = 9)	5.2 ± 1.2 (*n* = 5)	6.3 ± 0.9 (*n* = 6)

## Abbreviations

bTJ	Bicellular tight junction
d_(Å)_	Diameter (Å)
EDTA	Ethylenediaminetetraacetic acid
FD4	4 kDa-FITC-dextran 4 kDa
FITC	fluorescein isothiocyanate-dextran
KD	Knockdown
MDCK C7	Madin-Darby canine kidney cells, clone 7
shRNA	small hairpin RNA
TJ	Tight junction
tTJ	Tricellular tight junction
P	Apparent permeability (cm s^−1^)
PBS	phosphate-buffered saline
SEM	Standard error of the mean
TAMP	tight-junction-associated marvel protein
TER	Transepithelial resistance (Ω cm^2^)
